# Improvement of semantic processing ability of Chinese characters in school children: A comparative study based on 2009 and 2019 data

**DOI:** 10.3389/fnins.2023.1110674

**Published:** 2023-03-08

**Authors:** Qinfen Zhang, Xuan Dong, Yan Song, Chaoqun Wang, Shiyan Ji, Haitian Mei, Rui Wang

**Affiliations:** ^1^Children’s Health Research Center, Changzhou Children’s Hospital of Nantong University, Changzhou, Jiangsu, China; ^2^State Key Laboratory of Cognitive Neuroscience and Learning & IDG/McGovern Institute for Brain Research, Beijing Normal University, Beijing, China

**Keywords:** semantic processing, event-related potential, school children, development, environmental factors

## Abstract

To explore the characteristics of semantic cognitive development of school children by observing the development changes over 10 years, a retrospective event-related potential (ERP) study was conducted on the semantic processing characteristics of Chinese characters in children aged 7–11 years with the same study design in 2009 and 2019. For the EEGs recorded in 2009, the N400 amplitude of semantic processing in children aged 7–11 years showed an approximately inverted U-shaped development trend with a slow rise at the age of 7–9, a peak at the age of 10, then a rapid decline at the age of 11. However, for the EEGs recorded in 2019, the N400 amplitude showed a gradually decreasing development trend with a slow decline for the 7–11 years class. Our data suggested that the semantic processing of Chinese characters in children aged 7–11 years in 2019 was one age stage earlier than that in 2009. The children’s brain cognition is in the process of development and change with high plasticity. 10 years of favorable social and educational environmental factors have significantly improved children’s semantic processing ability of Chinese characters.

## Introduction

Language is one of the key functions of the human brain and plays an important role in communication. Children’s language development is closely linked to their understanding of the world and the shaping of their selves. Language is therefore a fundamental cognitive skill throughout life. The cortical language network can be roughly subdivided into two main developmental stages (Skeide and Friederici, 2016). In the first stage extending over the first 3 years of life, the infant rapidly acquires bottom-up processing capacities, which are primarily implemented bilaterally in the temporal cortices. In the second stage continuing into adolescence, top-down processes emerge gradually with the increasing functional selectivity and structural connectivity of the left inferior frontal cortex. In the second stage of language network development, school-age children are maturing in their ability to understand language. By the end of children’s first decade, they employ a similar cortical network for lexical semantic processes as adults, including activation in left inferior frontal, left middle temporal, and bilateral superior temporal gyri (Moore-Parks et al., 2010).

The understanding of language requires a well-orchestrated interplay of several cortical regions to segment the incoming auditory stream into words that can be associated with meaning (semantics) and combined into sentences following certain rules (syntax). Comprehension is achieved once linguistic meaning is successfully mapped onto and integrated into existing world knowledge. In the context of the cognitive model of auditory language comprehension, a first step toward comprehension is the access to the lexicon and the information encoded in the lexical entry. During lexical access, the processing system must first check whether the base unit it identifies is legal in the target language. If a match is found, access to the word in the lexicon can start and syntactic and semantic information can be retrieved (Davis and Gaskell, 2009). A number of brain regions have been reported to support semantic processes: the anterior temporal lobe, the posterior temporal and middle temporal gyrus, the angular gyrus, and BA 47/45 in the inferior frontal gyrus (Lau et al., 2008; Mesulam et al., 2015). At the neuronal level, neurons representing basic semantic features may be involved in various sets of different lexical representations, with semantically related words with overlapping semantic features leading to partially overlapping sets. This view is in principle consistent with a behavioral effect long known in the psychological literature as a priming effect, referring to the observation that semantically related words (cattle and horse) are easier to process when presented after a short delay. The process by which an external symbol, word or vocabulary enters the cognitive structure system and activates the corresponding word meaning in his mental lexicon and facilitates the association of the symbol, word or vocabulary with which it is associated is known as the semantic priming phenomenon. As stimulus information is presented, the individual’s ability to recognize and perceive this stimulus information increases. Its representation is more easily extracted relative to other stimulus information that has not been presented, and this is known as the semantic priming effect.

In recent years, event-related potential (ERP) (Lohvansuu et al., 2018), with the advantage of high temporal resolution, has greatly facilitated the study of language processing by recording the time course of word processing. N400 is an ERP component that is closely related to cognitive processing of language, and a large number of psychological studies of language are analyzed by means of the N400. N400 is sensitive to semantic priming (Weimer et al., 2019), and its amplitude is related to the difficulty of word retrieval (Delogu et al., 2019) and semantic integration (Berkum et al., 2009; Zunini et al., 2017). In essence, N400 is induced by the conflict with expectation (Kutas and Federmeier, 2000, 2011; Lau et al., 2008). The amplitude of the N400 effect in language processing cognition is related to the predictability of keywords. The more unpredictable the keywords are, the greater the amplitude of the N400 effect.

The N400 effect is a multi-source, multi-site action brain response, and the origin of the N400 is influenced by different experimental tasks and different stimulation modalities. At present, the interpretation of N400 is different (Junge et al., 2021). There are two main theories: diffusion activation (automatic activation process) and semantic integration. Some researchers believe that N400 reflects the processes associated with post-lexical integration of words into a given context (lexical post-processing), while others position the N400 component at the level of semantic access (lexical processing) (Wang and Yuan, 2008). Regardless of either theory, N400 has extensive sensitivity to semantic processing (Kutas and Federmeier, 2011). A large number of studies covering language processing, recognition memory, number processing, and face processing have expounded the role of N400, and N400 components have become an ideal indicator for studying word processing in children.

Semantic processing is the core of language understanding. This study focused on the semantic processing of single Chinese characters, and used the classical semantic priming paradigm to explore the characteristics of brain cognitive development at the neural level of Chinese character processing in Chinese children aged 7–11 years. Prominently, this study incorporates both the 2009 and 2019 N400 results of semantic processing of two cohorts of 7–11 children under the same research design. The study provided more insight into the developmental characteristics and influencing factors of children’s language cognition, thus drawing attention to the issues that need to be addressed in the next step of our research to enhance language cognition.

## Participants and methods

### Participants

Normal children aged 7–11 years from an ordinary primary school in Changzhou were selected by random number method. Fifteen children were excluded due to the fact that <60% of the valid trials were retained after data pre-processing for obvious head movement and other reasons. After matching, 192 children were finally included in the study, including 89 in 2009 and 103 in 2019, grouped by age (Table 1). The inclusion criteria were: normal or corrected visual acuity, native Chinese speakers, right-handed, and had not previously participated in such experiments. We observed language development in typically developing children. So all participants exhibited IQ ≥80 according to the Wechsler Intelligence Scale for Chinese Children-Revised (WISCC-R) (Wang et al., 2021; Li et al., 2023). Children diagnosed by a specialist with attention deficit hyperactivity disorder, autism, psychological disorders, intellectual disability, and a history of psychiatric disorders or brain damage were excluded. All subjects had parental consent and signed informed consent, and participated in the experiment voluntarily and could adhere to it. This study was approved by the Ethics Committee of Changzhou Children’s Hospital of Nantong University.

**TABLE 1:** Demographic information of children in the final sample.

		7-year-old	8-year-old	9-year-old	10-year-old	11-year-old	Main effect (*p*)		Interaction (*p*)
							Age group	Recording year	
Male /female	2009	8/9	12/7	10/11	9/9	9/7	0.353	0.240	0.845
	2019	13/8	14/7	13/6	8/12	14/8			
IQ	2009	98.58 ± 8.37	94.00 ± 6.77	97.21 ± 6.61	93.07 ± 6.76	93.10 ± 6.98	0.149	0.977	0.728
	2019	94.58 ± 17.83	95.23 ± 12.15	101.08 ± 10.19	93.20 ± 15.02	92.14 ± 7.94			
SES	2009	29.71 ± 3.68	31.17 ± 7.23	27.42 ± 6.96	29.18 ± 8.99	31.63 ± 4.65	0.710	0.322	0.130
	2019	29.94 ± 3.73	31.12 ± 5.53	31.35 ± 7.40	33.65 ± 3.81	28.58 ± 5.90			

### Stimuli, design, and procedure

The comparative study of time lag across 10 years was conducted in 2009 and 2019 under the premise of the same school, age, and experimental technology.

In this experiment, 390 Chinese characters were stimulated, including true and pseudowords, and the true words were from the first grade of primary school Chinese textbooks published by Jiangsu Education Press. There were 195 pairs of priming and target characters, and they were divided into three types of character pairs, namely, semantic relevant (e.g., 牛**-**马), semantic irrelevant (e.g., 土**-**书), and pseudowords (e.g., 石**-**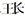); each of which had 65 pairs and appeared randomly in the ratio of 1:1:1. The prime word was the true word, the target word was the true or pseudo word, and the pseudo word conformed to the Chinese orthography rules. Before being selected as stimulus material, the characters were printed out on paper and distributed to students of each grade in several ordinary elementary schools. Only when the accuracy of semantic relevant, semantic irrelevant and pseudowords recognition is greater than 85%, those could be finally used in the experiment.

**FIGURE 1:**
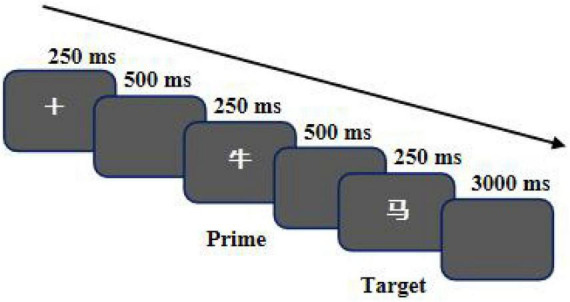
Temporal sequence of events in semantic priming task.

Participants sat 90 cm away from the computer screen, and both the vertical and horizontal viewing angles were 1.43°. The computer screen successively presented “+” for 250 ms, black screen for 500 ms, start word for 250 ms, black screen for 500 ms, and target word for 250 ms. Finally, the participants pressed the button and had a black screen for 3 s, and then entered the next stimulus group (Figure 1). Participants judged whether the target word was related to the priming word semantic meaning, and if relevant, pressed the right button, and if irrelevant, pressed the left button. At the same time, they checked whether the target word was a false word. If they judged that the target word was false, they did not press the button.

### ERP recording

The 32-guide Stellate digital paperless electroencephalography instrument was used. The reference electrode was CPz, and the eye movement recording electrode was located 2 cm above the left eye and 2 cm below the right eye. The scalp resistance was <5 kΩ, the amplifier bandpass was 0.1–70 Hz, and the sampling rate was 500 Hz. Before the test, children were asked to learn and understand the task, and the experiment was conducted after they fully understood the task.

### Data preprocessing

The German BESA software was used for off-line analysis to remove eye movements and other artifacts, and the filtering was conducted at 0.1–35 Hz. The average potential of bilateral ear lobes was re-referenced, baseline correction was made 200 ms before stimulation, and the analysis time was −200 to 1000 ms for target words. After pretreatment, the average effective trials ratio for each task was 81%.

### Statistical analysis

SPSS 25.0 statistical software was used in this study. With N400 amplitude as the dependent variable, 5 Age (7-, 8-, 9-, 10-, and 11 years-old children, between subject level) × 3 Stimulus Type (semantic relevant, semantic irrelevant, and pseudowords, within subject level) × 2 Recording Year (2009 and 2019 group, between subject level) with three-factor mixed design ANOVA was conducted.

## Results

The family Social Economical Status (SES) was compared, which was obtained from the parents’ educational level and occupation. A two-factor ANOVA with 5 Age (7-, 8-, 9-, 10- and 11 years-old) × 2 Recording Year (2009 and 2019 group) was conducted for gender, IQ, and SES, respectively. The main effects and interaction effect were not significant (in all cases, *p* > 0.05). Table 1 shows the demographic data.

**FIGURE 2:**
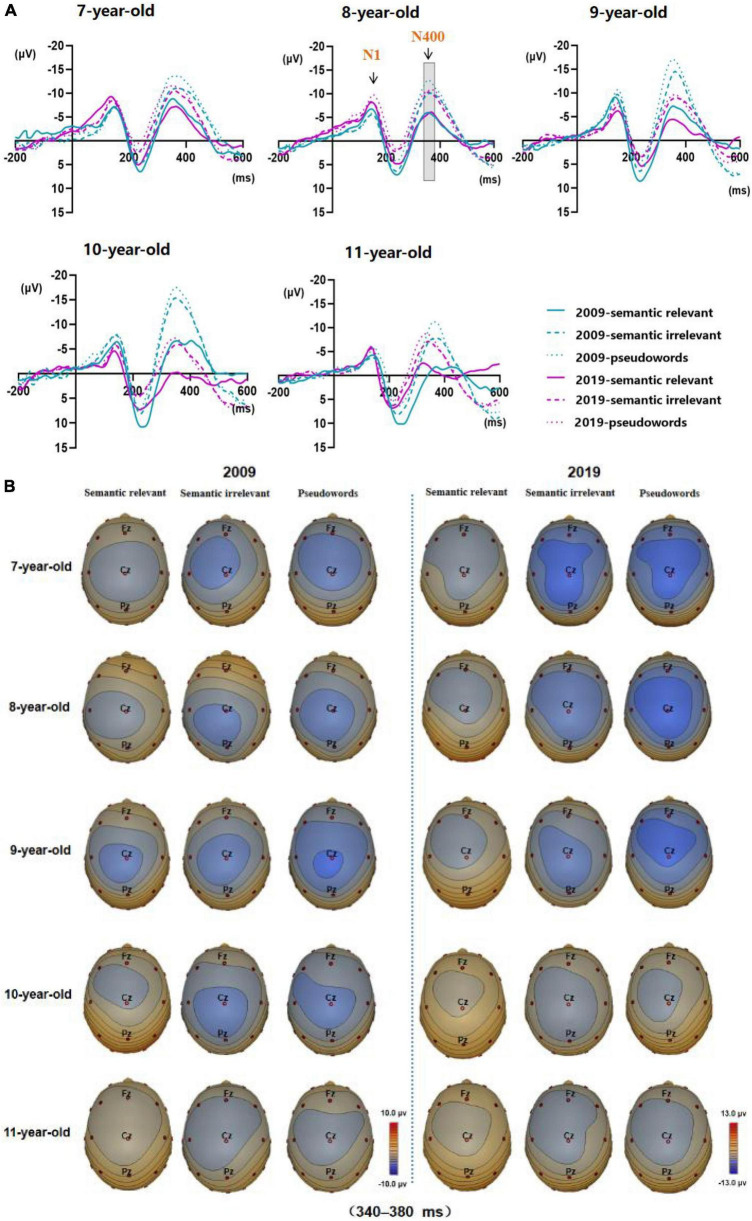
Event-related potential and topographic map results. **(A)** ERP waveforms at central electrode (Cz) of children aged 7–11 years in 2009 and 2019. The green wave is 2009, and the red is 2019. In addition, the solid line represents semantic relevant, the broken line represents semantic irrelevant, and the dashed line represents pseudowords. **(B)** Mean topographic map at 340–380 ms of children aged 7–11 years. Fz, Cz, and Pz electrodes are marked.

**FIGURE 3:**
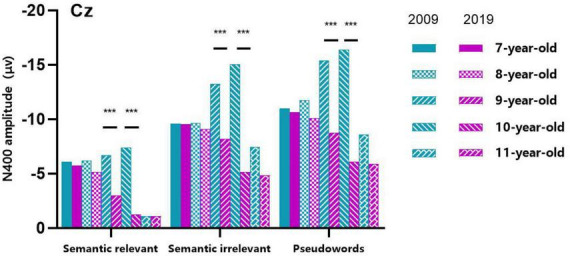
Comparison of N400 amplitude of children aged 7–11 years across 10 years. N400 amplitude of children aged 9 and 10 years in 2009 was significantly larger than that of the same age group in 2019. The symbol “***” represents *p* < 0.001.

The ERP waveforms and topographic map of Chinese character semantic processing for children aged 7–11 years in 2009 and 2019 are shown in Figure 2. Topographic maps have been analyzed for each age group of 7–11 year olds, and the results all indicated that Cz electrode should be concerned. Combined with the previous work of our team (Wang et al., 2021), this study focused on the Chinese character semantic processing N400 at Cz electrode, and the three-factor mixed design ANOVA was carried out in a 340–380 ms time window. Table 2 and Figure 3 show the N400 amplitude at Cz electrode of children in 2009 and 2019 recording groups.

**TABLE 2:** N400 amplitude of children in different years and age groups (*M* ± SD, μV).

Stimulus types		7-year-old	8-year-old	9-year-old	10-year-old	11-year-old
2009	Semantic relevant	−6.07 ± 3.11^b^	−6.19 ± 5.21^ad^	−6.65 ± 6.78^e^	−7.37 ± 7.52^e^	−1.33 ± 2.19^c^
	Semantic irrelevant	−9.57 ± 2.7^b^	−9.63 ± 6.36^ad^	−13.24 ± 6.95^e^	−15.03 ± 8.99^e^	−7.43 ± 4.88^c^
	Pseudowords	−10.95 ± 3.00^b^	−11.73 ± 5.55^ad^	−15.35 ± 9.61^e^	−16.39 ± 8.64^e^	−8.56 ± 3.81^c^
2019	Semantic relevant	−5.71 ± 3.99^fg^	−5.21 ± 5.09^fg^	−2.97 ± 3.48	−1.23 ± 3.67	−1.07 ± 2.96
	Semantic irrelevant	−9.54 ± 3.67^fg^	−9.09 ± 5.81^fg^	−8.19 ± 6.28	−5.12 ± 6.41	−4.84 ± 3.45
	Pseudowords	−10.6 ± 4.84^fg^	−10.07 ± 6.69^fg^	−8.70 ± 6.55	−6.08 ± 5.90	−5.86 ± 4.40

In 2009, compared with the 10-year-old group in the same year, ^a^*p* < 0.05, ^b^*p* < 0.01, ^c^*p* < 0.001; compared with the 11-year-old group in the same year, ^d^*p* < 0.05, ^e^*p* < 0.001. In 2019, compared with the 10-year-old group in the same year, ^f^*p* < 0.001; compared with the 11-year-old group in the same year, ^g^*p* < 0.001.

The three-way interaction among Recording Year, Age, and Stimulus Type was not significant (*F*_(8,531)_ = 0.309, *p* = 0.963). There was a significant main effect of Stimulus Type (*F*_(2,531)_ = 57.917, *p* < 0.001). After multiple comparisons, N400 amplitude was significantly different between semantic irrelevant and semantically relevant, and between pseudowords and semantic relevant (in all cases, *p* < 0.001). There was a marginal difference between semantic irrelevant and pseudowords (*p* = 0.094). The amplitude of N400 of semantic irrelevant and pseudowords was significantly larger than that of semantic relevant. These results were consistent with previous studies (Zhao et al., 2016), indicating the stability and reliability of the our EEG data and N400 is an important marker of semantic processing in children.

There were significant effects of Age (*F*_(4,531)_ = 10.176, *p* < 0.001) and Recording Year (*F*_(1,531)_ = 49.806, *p* < 0.001), and the significant interaction of Age with Recording Year (*F*_(4,531)_ = 10.197, *p* < 0.001). A simple effect of Age was significant in both groups of children (2009 group: *F*_(4,531)_ = 10.931, *p* < 0.001; 2019 group: *F*_(4,531)_ = 9.296, *p* < 0.001). Pairwise comparisons showed that, for the 2009 group, the N400 was significantly larger in 10 year-old children than in 7-, 8- and 11 year-old children (in all cases, *p* < 0.05), while the N400 was significantly smaller in 11 year-old children than in 8-, 9- and 10 year-old children (in all cases, *p* < 0.05). Therefore, the N400 amplitude recorded in the 2009 showed a slow rise at the age of 7–9, a peak at the age of 10, then a rapid decline at the age of 11. For the 2019 group, the N400 were significantly smaller in 10- and 11 year-old children than in 7- and 8 year-old children, respectively (in all cases, *p* < 0.05), and the N400 were marginally smaller in 11 year-old children than in 9 year-old children (*p* = 0.085). Therefore, N400 amplitude recorded in the 2019 decreased slowly at the age of 7–9 and dropped rapidly at the age of 10–11. In summary, our results showed that the development trend of N400 amplitude in children aged 7–11 years was particularly different between the 2009 and 2019 recording year groups (Figures 2, 3).

A simple effect test further showed that N400 amplitude of children aged 9 and 10 years in 2009 was significantly larger than that of the same age group in 2019 (9 year-old children: *F*_(1,531)_ = 24.369, *p* < 0.001; 10 year-old children: *F*_(1,531)_ = 61.594, *p* < 0.001). Whereas this effect did not reach significant at 7-, 8- and 11 years old children (in all cases, *p* > 0.132).

In order to eliminate the system error and equipment error between the 2009 and 2019 recording year groups, a three-factor mixed design ANOVA was conducted in the N1 amplitude at Cz electrode (100–180 ms) with 5 Age Classes (7, 8, 9, 10, and 11 years old, between subject level) × 3 Stimulus Type (semantic relevant, semantic irrelevant, and pseudowords, within subject level) × 2 Recording Year (2009 and 2019 group, between subject level). The results showed that the main effect of recording year was not significant (*F*_(1,531)_ = 0.332, *p* = 0.565), and the interaction of recording year with Age and Stimulus Type was not significant (*F*_(4,531)_ = 1.966, *p* = 0.098; *F*_(2,531)_ = 0.067, *p* = 0.935), and there was no interaction between the three factors (*F*_(8,531)_ = 0.254, *p* = 0.980). The main effect of Age was significant (*F*_(4,531)_ = 11.443, *p* < 0.001), which was consistent with previous studies that N1 decreased with children development (Amora et al., 2022). However, our results further showed that there was no significant difference in N1 amplitude between 2009 and 2019 recording group, suggesting that the basic visual processing development is similar between the two groups.

## Discussion

This study aimed to explore the semantic cognitive development of Chinese school children across 10 years by comparing the N400 amplitude. It was found that the semantic processing ability of Chinese characters of children aged 7–11 years in 2019 was significantly improved compared with that of 2009.

Semantic understanding is a complex cognitive psychological process based on learning and experience and is an important step in verbal communication. And semantic processing skills are one of the keys to semantic understanding. ERP can provide important information about cortical processing due to a highly time-locked relationship with semantic processing (Lu et al., 2016). N400 is induced by conflict with expectations (Kutas and Federmeier, 2000; Lau et al., 2008). In the semantic priming task, N400 amplitude decreases when the target word is semantically related to the priming word, or increases if semantically unrelated (Guo and Peng, 2003), as confirmed by our study. Furthermore, the more difficult the process of semantic processing and integration is, the greater the N400 amplitude. Our previous study (Zhang et al., 2022) showed that the decrease of N400 amplitude was closely related to the maturity of children’s semantic understanding. It was also found that the N400-P600 two-stage effect of Chinese character in school-age children aged 7–11 years showed significant age-specific features. As age increased, context-evoked preparatory attentional responses became more pronounced, and the expectation of the coming specific stimulus was stronger, resulting in greater violations and conflicts. Therefore the resources and energy required to retrieve and integrate the cognitive monitoring system increased significantly, with a rapid increase in the N400-P600 two-stage effect at age of 10 and 11.

With age the children’s brains gradually converge towards the efficient functional architecture of adults. Activation of core semantic processing areas gradually increases (Weiss-Croft and Baldeweg, 2015), and the strength of functional connectivity between the left inferior frontal gyrus triangle and the left temporal parietal area increases (Qi et al., 2021). As the activation of high-level control regions reduces, and the automaticity for language processing increases as children become more proficient. Our results showed that children’s semantic processing in 2009 and 2019 gradually matured with age in different developmental patterns. The development of semantic processing in children aged 7–11 years was recorded in 2009 in an approximately inverted U-shape, with the trend of N400 amplitude increasing slowly between the ages of 7–9 years, peaking at 10 years and decreasing rapidly at 11 years. This is similar to the development curve of the visual N170 component associated with expertise for visual stimuli such as words. Longitudinal studies have shown an inverted U-shape development curve of the N170, with an increased response for orthographic stimuli in beginning readers followed by a slight decrease when readers become fluent (Fraga-Gonzalez et al., 2021; Amora et al., 2022). Developmental data suggested an inverted U-shape trajectory of visual responses discriminating between words and symbols (Maurer et al., 2006). Children aged 7–9 years have a small store of knowledge and a simple understanding of semantics, and therefore do not require much anti-interference work, yet they may not truly grasp the semantics of Chinese characters. With the increase of age, 10 year-old children’s semantic understanding generates a diversity of ideas, and there is greater conflict inhibition in the analysis and recognition process, thus using more brain resources for the process. As further brain function improves, children at the age of 11 have established better semantic understanding and logical thinking skills. The way of thinking about problems is becoming more mature, the ability to discriminate is gradually increasing, and the semantic processing of Chinese characters is developing rapidly. In contrast, the N400 amplitude recorded in 2019 of children aged 7–11 years follows a trend of slowly decreasing at ages 7–9 and significantly decreasing at ages 10–11. Age of acquisition is the unique factors implicated in the processing of Chinese words (Xu et al., 2021). The older the child, the more knowledge storage and familiarity with the experimental material, and the more rational allocation of mental resources, so the amplitude becomes smaller.

The results of this study showed that children aged 9 and 10 in 2019 showed a significant decrease in N400 amplitude compared to children of the same age in 2009, suggesting a greater improvement in semantic processing function over the decade. We think that the trend in N400 amplitude for children aged 7–11 years recorded in 2019 has skipped the rising branch of 2009 and is only similar to the falling branch of 2009. The semantic processing development of children in 2009 and 2019 showed three stages: 7–9, 10, and 11 years old, while the position of 7–9 years olds in the development trend in 2019 was equivalent to that of 10 years olds in 2009. Based on this inference, the development of children’s semantic processing of Chinese characters in 2019 is one age stage earlier than in 2009. We focus more on the developmental curve within the group. Across 10 years, the developmental curves of semantic processing of children aged 7–11 years is different. The semantic processing ability of children in 2019 was significantly improved compared with that in 2009, especially in the group of 9 and 10 years old. The increase in gray matter volume of the frontal and parietal lobes peaks around age 10 (Paus, 2005). And executive abilities, such as working memory and response inhibition, also basically mature by the age of 10 (Giedd et al., 1999). Therefore, it is relatively easy for 11 year-old children to handle the three stimulus types of semantic processing, resulting in no statistical difference in the N400 amplitude of 11 year-old children between 2009 and 2019.

The results of the pseudowords in this study also confirm the improvement in children’s semantic processing skills. This lexical access process is suggested by a meta-analysis of brain imaging studies that compared the processing of real words with words that might be present in orthography in a language, but not in the lexicon of that language (Davis and Gaskell, 2009). Pseudowords elicit solid activation in the superior temporal gyrus close to the auditory cortex, indicating an initial search, and checking process for possible word forms. In contrast, real words elicit more activation in the anterior, posterior and inferior regions of the temporal cortex, reflecting the processes of lexical encoding and semantic extraction that follow the step of recognizing word forms (Friederici, 2017). Thus, the present study suggests that the process from lexical to semantic access was improved in the children recorded in 2019.

Brain cognition is the acquisition, processing, extraction, and output use of information input during human interaction with the environment. The environment is an important factor in the development of brain form and function. It can influence the structure and synaptic connectivity patterns of the brain’s complex neural networks by acquiring new experiences and activating or inhibiting genetic and molecular programs through sensory, motor, and emotional stimuli. The environmental factors that influence children’s brain development include social macro linguistic and cultural factors, family environment and schooling factors. The family educational environment contains a very large number of complex influencing variables, such as family economic income, parental education level, parental occupation, family structure, educational attitudes, parent–child interaction, and expectations of children. Family economic income, parental education level, and occupation are components of SES, which has a significant impact on children’s early phonological and vocabulary development (Zhang et al., 2013). It jeopardizes children’s cognition, learning ability, and physical and mental health, and may persist into adulthood and even affect lifelong development (Zhu et al., 2019; Tian et al., 2021). Educational attitudes, parent–child interaction and expectations of children are closely related to SES. The schooling environment encompasses the pre-school early childhood institutional setting and includes factors such as educational philosophy, educational methods and teaching environment. Early learning experiences from the school environment can help children improve their academic performance and build learning confidence.

Children aged 7–11 years attending the same general primary school in 2009 and 2019 were recruited for this study, and there were no differences in gender or intelligence (Wu et al., 2013) between the 2 years. Meanwhile, the SES of the families was analyzed, which was strongly associated with children’s language development (Brito and Noble, 2018; Su et al., 2021). One study showed no correlation between family income and language-related resting-state functional connectivity (Su et al., 2021). Family income is less accurate in the survey process, whereas parental education and occupation factors are relatively stable and reflect the basic income level of the family, so we used parental education and occupation to obtain the SES score by weighting. Our statistical calculations suggested that there are no significant differences in the SES of the families of the children included in the study between the 2 years, therefore the family environment factors were basically similar.

We proposed that the semantic processing of Chinese characters in children aged 7–11 years in 2019 has improved by one age stage compared with 2009. From the perspective of environmental factors in the development of brain cognition, we believe that the language and cultural factors play a large role, including the development of politics, economics, culture, science, and technology. Neuropsychological assessment needs to consider the children’s cultural background (Rosenqvist et al., 2017). China’s rapid socio-economic development is well known, and the implementation of the strategy of rejuvenating the country through science and education has promoted rapid development of science, technology, and education. In this environment, national cultural connotation has been enhanced and children’s semantic comprehension has improved compared to a decade ago. In addition, although the school-aged children participating in the study in 2009 and 2019 were from the same general primary school, the continuous progress in school education philosophy and reform in education methods would also promote the improvement of semantic cognition among school-aged children. Thus, the rapid development of the social environment and education has led to a significant increase in children’s language cognition, which also suggests that children’s brains are in a state of developmental change and are highly plastic.

Our study found no change in the underlying understanding of semantic relevance, irrelevance, and pseudowords of school-age children between 2009 and 2019. Firstly, the children were identifying true words and pseudowords (Davis and Gaskell, 2009), in which the false words did not meet expectations and therefore triggered larger-amplitude N400 responses. Secondly, the true word required further judgment of whether there was a semantic correlation. Finally, if no correlation, it was a semantic irrelevance, which triggered a larger amplitude N400 component than correlation. Thus the pseudowords and semantic irrelevance induced larger-amplitude N400 responses than semantic relevance. In addition, the Chinese characters used in our study are high-frequency or sub-high-frequency characters in the Chinese textbooks of the first grade of primary school, which are easy to understand in terms of semantic relevance (Liu et al., 2019; Wang and Zhang, 2021). However, the semantically unrelated processing is more difficult because there are more conflicts and integration of semantic features between Chinese characters. Compared with true words, pseudowords have no corresponding representation in the mental dictionary, so it is difficult to identify them. These results can explain the differences among children in semantic related, unrelated and pseudowords. In our study, the differences in these three kinds of semantic processing cognitive ability were stable in children aged 7–11 years, both in 2009 and 2019, which confirmed the stable repeatability of our experimental task. There was no significant difference in N1 wave between 2009 and 2019 among children aged 7–11 years, which can also exclude the error caused by the equipment.

## Conclusion

In conclusion, children’s semantic processing ability develops at a non-uniform rate with age, and it is modified and adjusted in the process of interaction with environmental factors. With the development of society and the progress of education, children’s language cognition has been significantly improved. The cognitive processing of Chinese characters is complex, and N400 plays an important role in revealing its deep brain mechanism.

The research had some limitations. The behavioral performance in semantic processing tasks of the children were not reported in this study, because part of the behavioral data in 2009 was not recorded. Our data management needs to be strengthened in larger studies over long periods of time. The current study focuses on the comparison of N400, and we will report other components such as N170, P2, and P600 in other articles. In terms of technological method, if ERP can be combined with functional magnetic resonance imaging (fMRI) and other technologies, their respective advantages will be brought into play. We will better investigate the developmental characteristics of primary school students’ Chinese cognition to provide scientific basis for translation and application.

## Data availability statement

The datasets presented in this study can be found in online repositories. The names of the repository/repositories and accession number(s) can be found below: The data for the present study are available through the Open Science Framework (https://osf.io/hpmzb/).

## Ethics statement

The studies involving human participants were reviewed and approved by the Ethics Committee of Changzhou Children’s Hospital of Nantong University. Written informed consent to participate in this study was provided by the participants’ legal guardian/next of kin.

## Author contributions

QZ: supervision, conceptualization, methodology, formal analysis, investigation, writing—original draft, and visualization. XD: conceptualization, methodology, and writing—review and editing. YS: conceptualization, methodology, and writing modification. CW: conceptualization, writing—original draft, and investigation. SJ and RW: conceptualization, formal analysis, and investigation. HM: conceptualization, software, formal analysis, and investigation. All authors contributed to the article and approved the submitted version.
